# The Population Diversity of Candidate Genes for Resistance/Susceptibility to Coronavirus Infection in Domestic Cats: An Inter-Breed Comparison

**DOI:** 10.3390/pathogens10060778

**Published:** 2021-06-21

**Authors:** Jana Bubenikova, Leona Vychodilova, Karla Stejskalova, Jan Futas, Jan Oppelt, Petra Cerna, Martin Plasil, Petr Horin

**Affiliations:** 1 Department of Animal Genetics, Faculty of Veterinary Medicine, University of Veterinary Sciences Brno, 61242 Brno, Czech Republic; bubenikovaj@vfu.cz (J.B.); vychodiloval@vfu.cz (L.V.); stejskalovak@vfu.cz (K.S.); jfutas@vfu.cz (J.F.); 2 CEITEC UVS, RG Animal Immunogenomics, University of Veterinary Sciences Brno, 61242 Brno, Czech Republic; jan.oppelt@gmail.com (J.O.); plasilma@vfu.cz (M.P.); 3 Department of Pathology and Laboratory Medicine, Division of Neuropathology, Perelman School of Medicine, University of Pennsylvania, Philadelphia, PA 19104-6100, USA; 4 Department of Clinical Sciences, Colorado State University, Fort Collins, CO 80523-1678, USA; petra.cerna@colostate.edu

**Keywords:** feline coronavirus (FCoV), interbreed differences, SNP, candidate genes, principal coordinate analysis (PCoA)

## Abstract

Feline coronavirus (FCoV) is a complex pathogen causing feline infectious peritonitis (FIP). Host genetics represents a factor contributing to the pathogenesis of the disease. Differential susceptibility of various breeds to FIP was reported with controversial results. The objective of this study was to compare the genetic diversity of different breeds on a panel of candidate genes potentially affecting FCoV infection. One hundred thirteen cats of six breeds were genotyped on a panel of sixteen candidate genes. SNP allelic/haplotype frequencies were calculated; pairwise FST and molecular variance analyses were performed. Principal coordinate (PCoA) and STRUCTURE analyses were used to infer population structure. Interbreed differences in allele frequencies were observed. PCoA analysis performed for all genes of the panel indicated no population substructure. In contrast to the full marker set, PCoA of SNP markers associated with FCoV shedding (*NCR1* and *SLX4IP)* showed three clusters containing only alleles associated with susceptibility to FCoV shedding, homozygotes and heterozygotes for the susceptibility alleles, and all three genotypes, respectively. Each cluster contained cats of multiple breeds. Three clusters of haplotypes were identified by PCoA, two clusters by STRUCTURE. Haplotypes of a single gene (*SNX5)* differed significantly between the PCoA clusters.

## 1. Introduction

Feline coronavirus (FCoV) is primarily a pathogen of the gastrointestinal tract of domestic cats. The virus is a member of the family *Coronaviridae*, belonging to the genus *Alphacoronavirus*. It is related to canine enteric coronavirus and transmissible gastroenteritis virus of pigs [1]. It is a complex pathogen, existing as two biotypes. Feline enteric coronavirus (FECV) can cause subclinical disease. Feline infectious peritonitis virus (FIPV) is a virulent and highly pathogenic form, causing a fatal clinical disease, feline infectious peritonitis (FIP). Only a small proportion of cats exposed to FCoV develop FIP [2]. The transition between the two viral biotypes was reported to be due to mutations in the FECV genome resulting in changes of the tropism and pathogenicity of the agent [3]. However, some recent findings suggest that the complexity of the virus has not yet been fully understood [4,5].

Similarly, there are important gaps in the knowledge of the pathogenesis of this rather enigmatic disease. In general, the properties of the virus, the reaction of the host organism, and environment play a role in the mechanisms of disease. Host factors important in the development of FIP include age, genetics, and immune system status, along with concurrent disease and environmental stress, both of which can affect immune responses. The immune system is known to play a critical and complex role in the pathogenesis of FIP; however, that role is still incompletely understood [6].

Since FECV infection is primarily spread by the orofecal route [7], the role of the immune system is important not only in responses to FIPV infection, but also in the control of fecal shedding of the virus. Shedding usually begins within one week of exposure and cats may shed FCoV with quite a variable intensity in some cats, FCoV shedding may persist for more than 18 months [7]. Individual variation in the patterns of fecal shedding of FCoV was observed. Asymptomatic persistent shedders represent the most important source of infection [8].

Host genetics represents one of the factors contributing to the observed variation in immune responses to coronavirus infections, both in humans and animals [9]. Two major phenotypes associated with FCoV infections can be distinguished: clinical disease (FIP) and fecal FCoV shedding. As for FIP, a comprehensive description of the role of host genetics in its pathogenesis is still lacking, despite general agreement on its importance. Differential susceptibility of various breeds and lines to FIP was repeatedly observed; pedigree cats were reported to be more susceptible to FIP than non-purebred cats [3,10,11]. However, as different populations of the same breed were analyzed and different diagnostic methods were used, the data are not always comparable. It is not even clear whether the data published so far apply for all populations within a breed. Moreover, for many breeds, it is not clear from epidemiological studies whether their “resistant” status might in reality be due to a low infectious pressure in the studied population. Interbreed differences in the presence of anti-FCoV antibodies in healthy cats have also been reported [8,12,13]. It is not clear to what extent this might be due to non-genetic factors, such as the intensity of close contacts between pedigree and non-purebred cats.

Statistical associations of candidate immune response (IR) genes, such as interferon-gamma and tumor necrosis factor alpha coding genes, with individual variability in clinical FIP status were analyzed with sometimes controversial results [3,14,15,16]. Two GWAS studies were performed, resulting in a definition of candidate regions associated with FIP [10,17]. The most relevant finding was a strong association identified for an 11.1 Mb long region of chromosome A3. However, no candidate genes were found in this region [17].

While attention has been paid to the role of IR genes in individual susceptibility to FIP, little is known about their role in individual variation of intensity and patterns of FCoV fecal shedding, despite its epidemiological importance. Therefore, only limited and rather fragmentary information on the role of host genetics in the observed individual variability of FCoV fecal shedding is available. A study of molecular markers on a limited number of cats did not find statistical associations between FCoV fecal shedding status and the feline Major Histocompatibility Complex (MHC) FLA class II DRB genes [18]. Recently, we have found associations between SNPs of one functional and one positional candidate gene (*NCR1* and *SLX4IP*, respectively) and haplotypes of four genes (*SNX5, NCR2, SLX4IP, NCR1*) with FCoV shedding in a multiple-breed study [19].

In general, the currently available data on inter-breed and within-breed (individual) genetic variation are not sufficient to distinguish between their relative contributions to the overall phenotypic variability of coronavirus infections observed in domestic cats. If confirmed, strong phenotype/genotype associations may be considered to identify individual cats susceptible to FIP and/or to massive virus shedding by genetic testing. However, for this purpose, it is important to determine to what extent they are breed-specific and/or valid across breeds.

The objective of this study was to compare the genetic diversity of different breed groups on a panel of candidate immune response genes potentially affecting FCoV shedding, with a special focus on genes associated with FCoV shedding in our previous study.

## 2. Results

### 2.1. Within-Breed Diversity of Candidate Genes

A total of 16 genes were analyzed in all breed groups. Total numbers of SNPs and other polymorphisms identified in all groups are in Supplementary Table ST1.

Parameters of genetic diversity for all genes are in Supplementary Material SM1. Based on the average number of SNPs per 1 kb, the most polymorphic sequence across all breeds was *PRF1*, while the least polymorphic sequence was *SLX4IP*. The most diverse group was Domestic Shorthair stray cats, the least diverse group was Russian Blue (Supplementary Table ST1).

### 2.2. Inter-Breed Comparisons

Interbreed comparisons of allele frequencies for all genes analyzed are in Supplementary Table ST2. For each gene, out of 15 possible comparisons between two breeds, six to fourteen differed significantly (*p*_corrected_ < 0.01) in their allele frequencies (Table 1).

### 2.3. FST-Based Comparisons, PCoA and STRUCTURE Analyses

Comparisons including all candidate genes and comparisons made for genes associated with fecal shedding at *p*_uncorrected_ < 0.01 and at *p* > 0.05 in our previous study [19] revealed specific clustering patterns of genes associated at *p*_corrected_ < 0.01.

#### 2.3.1. FST-Based Comparisons

Pairwise FST values based on all analyzed genes indicated different relationships among the breeds of the panel (Table 2). Pairwise FST values based on genes associated with FCoV shedding at *p*_corrected_ < 0.01 (Table 2) suggest higher levels of genetic differentiation between Bengal cats and Russian Blue cats (FST 0.413). These two breeds seem to be genetically distinct when compared with other populations. All pairwise FST values calculated separately for individual genes associated with FCoV shedding at *p*_corrected_ < 0.01 and other analyzed groups of markers are in Supplementary Material SM2.

#### 2.3.2. PCoA and STRUCTURE Analyses

**Figure 1:**
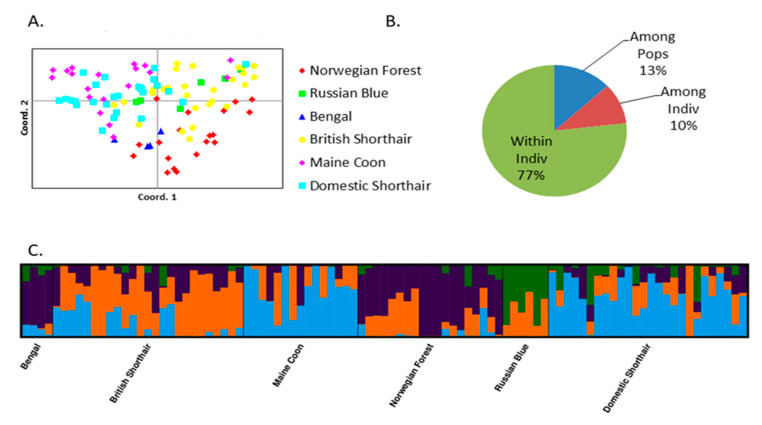
Results of PCoA, AMOVA and STRUCTURE analyses of cat breeds based on all 16 analyzed genes. (**A**) Principal coordinate analysis plots (first and second coordinate, 16 genes); (**B**) Molecular variance analysis (16 genes); (**C**) Estimate of the population structure across all 16 genes analyzed based on a STRUCTURE barplot (K = 4) sorted by predefined populations.

The results of the PCoA analysis performed for all genes of the panel indicated no population substructure. Although some breeds could be distinguished, they did not form separate clusters (Figure 1A). The first two axes in the PCoA plot explained 19.67% of variation. The analysis of molecular variance (AMOVA) revealed more genetic variance between individuals (77%) compared to variance between populations (13%) (Figure 1B). The optimal cluster number in STRUCTURE results based on delta K was 4. The Russian Blue cats showed a different pattern of clustering compared to the rest of breeds (Figure 1C).

**Figure 2:**
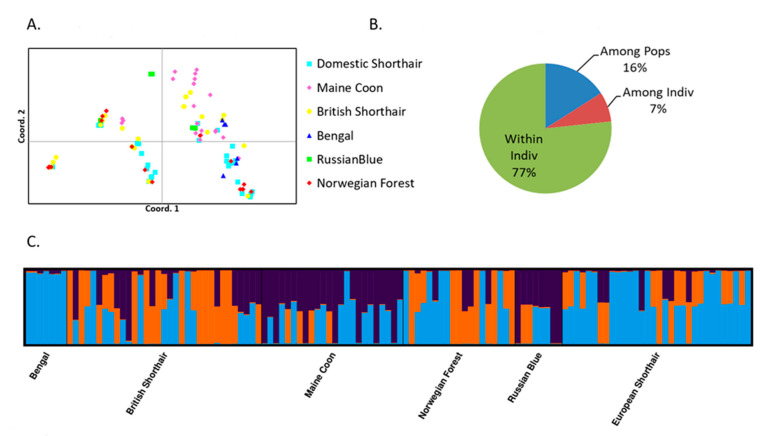
Results of PCoA, AMOVA and STRUCTURE analyses of cat breeds based on two genes associated with FCoV shedding at *p*_corrected_ < 0.01. (**A**) Principal coordinate analysis plots (first and second coordinate, two genes); (**B**) Molecular variance analysis (two genes); (**C**) Estimate of the population structure across two genes associated with FCoV shedding at *p*_corrected_ < 0.01 based on a STRUCTURE barplot (K = 3) sorted by predefined populations.

When markers were analyzed based on their associations with shedding, PCoA of two associated markers at *p*_corrected_ < 0.01 showed clearly separated groups (Figure 2A). Principal coordinates explained 58.96% of genetic variation; the first and second coordinates revealed 38.96% and 20% of the variation, respectively. The analysis of molecular variance revealed 77% genetic variance between individuals (Figure 2B). The results of the STRUCTURE analysis of associated genes are in agreement with the PCoA data. The optimal number of clusters based on delta K was K = 3 (Figure 2C).

For PCoA of individual markers associated at *p*_corrected_ < 0.01 (*NCR1* and *SLX4IP*), a substructure was also observed (Supplementary Material SM2). However, no trend to sub-structuring was observed for markers associated at *p*_uncorrected_ < 0.01 and for non-associated markers (Supplementary Material SM2).

A statistical comparison of allelic frequencies among the three clusters defined for associated genes showed significant differences between the clusters, including SNPs associated with FCoV shedding (Table 3, Supplementary Material SM3). The three clusters, respectively, contained only alleles of both genes associated with susceptibility to FCoV shedding in a homozygous constitution (Cluster 3), homozygotes and heterozygotes for the susceptibility alleles (Cluster 2), and all three genotypes (Cluster 1). The clusters did not distinguish breeds, with each cluster containing cats of at least three breeds. Bengal cats were the only breed found within a single cluster (Figure 2A). Overall differences in allelic frequencies and homozygosity between the clusters identified by PCoA expressed as numbers of SNPs, for which statistically significant differences were observed, are in Table 3.

### 2.4. Haplotypes

**Figure 3:**
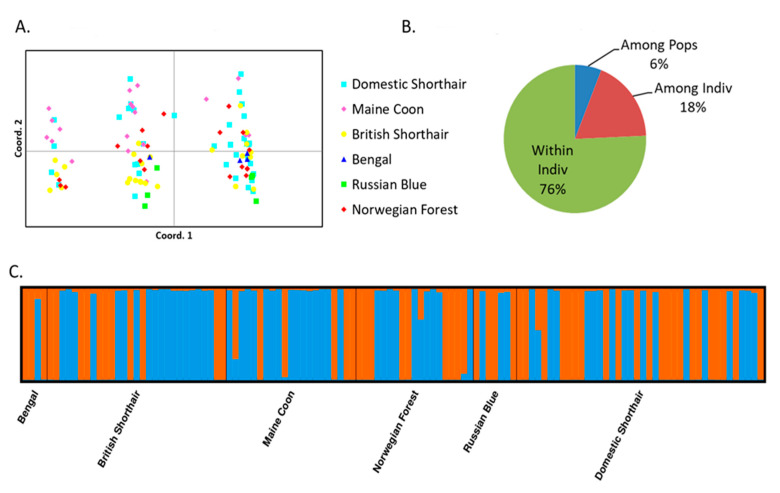
Results of PCoA, AMOVA and STRUCTURE analysis of cat populations based on haplotypes associated with FCoV shedding. (**A**) Principal coordinate analysis plots (first and second coordinate, 9 haplotypes of 4 genes associated with FCoV shedding). (**B**) Molecular variance analysis of 9 haplotypes of 4 genes associated with FCoV shedding. (**C**) Estimate of the population structure across 9 haplotypes of 4 genes associated with FCoV shedding based on a STRUCTURE barplot (K = 2) sorted by predefined populations.

Population parameters of nine haplotypes (*NCR1-D*, *NCR2-M/A*, *NCR2-S*, *SLX4IP-A*, *SNX5-A*, *SNX5-B*, *SNX5-C*, *SNX5-D*, *SNX5-E*) associated with FCoV shedding in our previous study [19] are summarized in Supplementary Material SM4. Data for all haplotypes analyzed are in Supplementary Table ST3. Out of all haplotypes generated by NGS and assessed by physical phasing, only one per gene contained a FCoV-associated SNP. Interbreed comparisons of frequencies made for these particular haplotypes (*SLX4IP-A*, *NCR1-D*) are in Table 4. For these haplotypes, inter-individual variation accounted for 76% of genetic variance, while variance among populations accounted for only 6% (Figure 3B).

**Figure 4:**
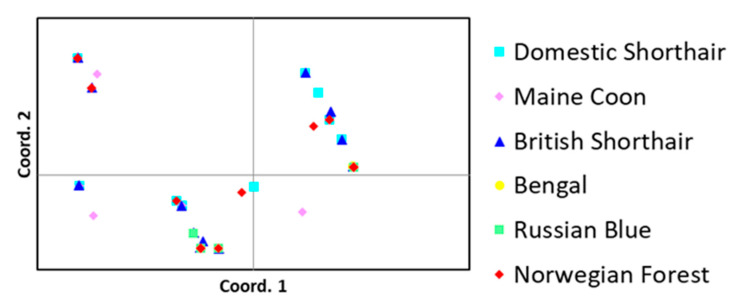
Principal coordinate analysis of cat breeds based on *SNX5* gene haplotypes.

Three clusters were observed by PCoA, while two clusters were identified by STRUCTURE (Figure 3). Analysis of differences in haplotype frequencies revealed that only haplotypes of *SNX5* differed significantly between the clusters identified (Supplementary Material SM5). A similar pattern was then found by PCoA performed for *SNX5* alone (Figure 4).

Principal coordinates explained 81.34% of genetic variation; the first and second coordinates revealed 70.54% and 10.80% of variation, respectively.

## 3. Discussion

This study is a follow-up of our previous association analysis of FCoV fecal shedding. Our panel of markers covered functional and/or positional candidates potentially associated with both clinical disease (FIP) and fecal shedding. Ten of these markers were fully characterized in this report [19]. Three IR gene markers were developed as functional candidates based on the roles of perforin (PRF1), granzyme B (GZMB) and granzyme A (GZMA) in the cytotoxicity of natural killer cells and T lymphocytes. These molecules seem crucial in developing systemic spread of virus and FIP development [20]. Considering the possible role of stress on FCoV pathogenesis [21,22,23], genes coding for molecules involved in stress reactions were added to the marker set as functional candidates. *CRH* encodes the preprotein of the neuropeptide hormone CRH (corticotropin releasing hormone). In response to stress, CRH is secreted from the hypothalamus and binds to its receptors (encoded by *CRHR1*) to activate the hypothalamic-pituitary-adrenal (HPA) axis. The CRH-binding protein (encoded by *CRHBP*) can modify the function of the HPA axis by binding to CRH [24], which leads to its inactivation.

To address the controversial issue of the role of breeds in FCoV infection and outcomes, the hypothesis tested in this study was that for the potentially non-neutral markers mentioned above, genetically more resistant and more susceptible breeds would form two clusters, each comprising similar breeds with regard to FCoV susceptibility/resistance. For this purpose, breeds reported as more resistant (Domestic Shorthair, Russian Blue, Maine Coon) and more susceptible to FIP (British Shorthair, Bengal, Norwegian Forest) [25,26,27] were analyzed. Based on the data obtained, this hypothesis did not prove to be true. When all markers were analyzed, only 13% of the variance could be attributed to variation among populations and 77% was due to variation among individuals (Figure 1). In agreement with this finding, PCoA and STRUCTURE analyses did not reveal any substructure, despite a non-random selection of markers for the panel. On the other hand, interbreed differences in minor allele frequencies (MAFs), most likely reflecting overall genetic differences between breeds, were observed for individual markers (Supplementary material SM1). Since the selection of cats for our study was restricted to breeders willing to participate in the study, we were not always able to obtain samples from unrelated cats, and for some cats, full pedigree information was not available. Due to inbreeding and (in some cases) small population sizes, a high level of overall homozygosity is observed in most established cat pure breeds anyway, as illustrated by our group of Russian Blue cats. In this breed, 75–95% of SNPs observed in the gene panel were homozygous, although only 5 out of 13 cats were related as sibs and/or half sibs. For Russian Blue and Bengal cats, small numbers of cats and higher homozygosity reflect the current breeding situation in these breeds, much less represented than ‘popular’ breeds with a broader genetic base such as Maine Coons, British Shorthairs and Norwegian Forrest cats. However, we do not think that this fact affects our interpretation of the data. Groups of closely related animals would be expected to form clusters separate from other cats of the breed and from other breeds in the PCoA, which was not observed. Cats of the same breed were always interspersed among other cats of other breeds, except for Bengals, which formed a small group of seven cats in PCoA and STRUCTURE. Some breeds were more isolated from the others (e.g., Norwegian Forrest, Bengal), but in general, breeds did not form separate clusters. As expected, the group of stray cats representing the European population of Domestic Shorthairs was the most diverse, comprising most of the variation observed within pure breeds. We have observed no differentiation between this group and purebred cats in this panel of candidate genes. Taken together, within the cohort analyzed, it was not possible to distinguish substructures indicating differentiation among breeds and/or between breeds based on the markers analyzed. In agreement with these data, a large proportion of the observed variation was due to variation among individuals, not among populations (Figure 1B).

There are two possible explanations for this finding. In the first place, our marker set could be non-informative for revealing such a substructure. Although the breeds involved were reported as more resistant or more susceptible to FIP in the literature, literary data on associations of various markers with clinical FIP are not fully consistent [3,14,15,16], and there is no experimental evidence on associations of the markers with clinical phenotypes. Besides this explanation, it is possible that even an informative marker set would produce the same data. This possibility is consistent with the large proportion of molecular variance attributable to differences between individuals as well as with the fact that PCoA and STRUCTURE analyses performed for markers associated with fecal shedding did not show significant interbreed differences.

On the other hand, the association of genes with FCoV shedding at different levels of significance [19] allowed us to analyze different types of markers separately and to include haplotypes. For SNP markers of two genes associated with FCoV shedding at *p*_corrected_ < 0.01, three clearly distinct clusters differing in the presence and frequencies of alleles associated with FCoV shedding were defined by PCoA and confirmed by STRUCTURE (Figure 2A,C; Table 3; Supplementary Material SM3). Non-randomly, two of the three clusters contained alleles associated with susceptibility to FCoV shedding in homozygous (Cluster 3) or both homozygous and heterozygous (Cluster 2) constitutions, while Cluster 1 contained all three genotypes. The clusters did not distinguish breeds; each cluster contained cats of at least three breeds. Bengal cats were the only breed found within a single cluster (Figure 2A). Thus, the cohort was clearly structured according to the presence of resistance/susceptibility-associated alleles, but not according to breeds. This finding was specific to the two genes associated with FCoV shedding, i.e., for no other group of markers was such clustering observed (Figures S1 and S3, Supplementary Material SM2). The PCoA pattern observed for the two genes individually also distinguished similar clusters (Figures S5 and S7, Supplementary Material SM2). These data are in agreement with the high proportion of variation of the associated genes among individuals compared to variation among populations (Figure 2B).

It is an interesting finding that PCoA and STRUCTURE analyses could distinguish between these three groups differing non-randomly in SNPs associated with FCoV shedding. The interpretation of this finding must consider some potential limitations. Only two genes were associated with shedding at *p* < 0.01 and could be included in this analysis. Nevertheless, the clusters identified by PCoA and supported by STRUCTURE contained non-randomly distributed alleles of the two loci. For both loci, *NCR1* and *SLX4IP*, each cat in Clusters 1 and 2 had at least one allele associated with susceptibility to FCoV shedding; their frequencies were significantly different between all three clusters (Supplementary Material SM3; Table 3). PCoA of other two-gene combinations, randomly selected among non-associated markers, showed no substructure (Supplementary Material SM2). We also must consider that one third (33 out of 113) of the cats of various breeds were individuals from the cohort used for the original association analysis. However, these cats were selected randomly for the original as well as for the new study. In both cases, they were chosen based on their breed, and not on their FCoV shedding phenotype. Two thirds of the cohort comprised cats for which fecal FCoV shedding phenotypes were not assessed. We thus think that the reuse of some cats cannot be the cause of the observed clustering patterns and that the clusters reflect individual genetic variability, which can be statistically associated with the previously studied phenotypes. There is a biological background for the speculation that some kind of selection pressure could have created genetically resistant and susceptible sub-populations across breeds exposed to FCoV. *NCR1* is expressed in NK cells, plays important roles in human coronavirus infections of zoonotic origin [28], and is probably involved in FIPV infection of cats [20,29]. *SLX4IP* was primarily a positional candidate, selected based on previously reported associations of the chromosome region where it is located with clinical FIP [17], and later also showing significant associations with FCoV shedder phenotypes [19]. We also may speculate that at least some immune mechanisms are common for the control of FCoV shedding and immunity to FIPV infection, and that a genetic differentiation involving multiple genes and their SNPs occurred under the selective pressure of FCoV, which is common in cat populations. The three clusters would then represent different groups composed of cats with different proportions of susceptibility-associated alleles, not only in the genes studied but also in other ignored genes, with Cluster 3 being the most heterogenous group. 

The clustering observed in the analysis of the two SNP markers associated with FCoV shedding (*NCR1* and *SLX4IP*) was not supported by the analysis of haplotypes. Nine haplotypes of 4 genes were significantly associated with FCoV shedding. However, PCoA and STRUCTURE analyses of these haplotypes did not show a consistent clustering pattern: three clusters were observed using PCoA, but only two clusters were identified by STRUCTURE (Figure 3A,C). Moreover, statistically significant differences in haplotype frequencies between the clusters were found only for haplotypes of *SNX5*. A similar pattern was also found by PCoA performed for *SNX5* alone (Figure 4), indicating that clustering based on all haplotypes was primarily determined by differences in *SNX5*. Thus, in contrast to the findings with respect to the two shedding-associated SNP markers, it was not possible to describe a substructure distinguishing resistant and susceptible genotypes based on shedding-associated haplotypes.

The difference between patterns observed for SNP and haplotype markers could be due to the fact that based on the short-read NGS technique, haplotypes could not always be purposefully selected, but rather were generated based on sequence coverage over a sequence of maximum length 200-250 bp. Therefore, the haplotypes studied did not represent a coherent set of markers. On the other hand, statistically inferred haplotypes were not considered to be suitable for another statistical analysis. Another factor potentially contributing to the difference between data based on SNPs and haplotypes is that haplotype combinations might differ according to breeds and bring additional variation to the analyses.

Altogether, the data showed that in contrast to analyses involving the full marker set, a population analysis based on two genes associated with FCoV shedding allowed us to distinguish clusters differing in frequencies of alleles previously associated with FCoV shedding phenotypes. It is not clear how these data may apply to FIP phenotypes. For both *NCR1* and *SLX4IP*, associations with FIPV infection are biologically plausible; however, for a similar analysis involving a multiple marker panel, genes associated with clinical FIP must be included. This is our task in the near future.

## 4. Materials and Methods

### 4.1. Cats

One hundred thirteen cats of five breeds (Maine Coon, Norwegian Forest, British Shorthair, Bengal, and Russian Blue) and 38 unrelated stray cats identified as Domestic Shorthairs were studied. Sixteen cats of 3 breeds were identified as relatives, while 39 purebred cats along with 38 stray cats were considered unrelated, and pedigree information was not available for the remaining cats (Supplementary Table ST4). Thirty-three purebred cats (15 Maine Coons, 14 British Shorthairs, 1 Bengal and 3 Norwegian Forrest) were phenotyped for FCoV shedding in our previous study [19]. No breed differences in shedder phenotypes were observed between the two breeds prevailing in this transferred group. All owners involved in the study signed an informed consent document and agreed to all related procedures.

### 4.2. Candidate Genes

Sixteen genes were selected as functional (***IFNL1, IFNLR1, NCR1, NCR2, NCR3****, PRF1, GZMA, GZMB, CRH, CRHR1, CRHBP*) and positional/functional (***SNX5, SLX4IP, PCSK2, PAK5, LAMP5***) candidates (Supplementary Table ST5). The functional candidates were selected based on their possible biological role in FCoV infection. Genes located within the region identified by the original GWAS study [17] with predicted immunity-related functions were selected as positional/functional candidates. Eight of the candidate genes are immunity related (*IFNL1, IFNLR1, NCR1, NCR2, NCR3, PRF1, GZMA, GZMB*) and three of the genes are connected with stress reactions (*CRH, CRHR1, CRHBP*). Genes **in bold** were studied for their associations with FCoV shedding in our previous study [19].

### 4.3. Genotyping

All candidate genes were genotyped as described in detail previously [19]. Briefly, the most recent domestic cat genome assembly (*Felis catus*, ver. 9.0, GenBank accession GCA_000181335.4) was used for identifying sequences corresponding to selected genes. The sequences then served for designing primers amplifying either full length genes or their functionally important parts (Supplementary Tables ST6 and ST7).

Peripheral blood was collected from all cats included in the study. Based on the informed consent signed by all owners, blood samples were always collected by a licensed veterinarian in agreement with all legal, professional and ethical standards, mostly at the occasion of vaccination and/or other veterinary interventions during the one-year study. This approach was approved by the Ethical Committee of the Veterinary University Brno. DNA was extracted from 200 μL of the EDTA-anticoagulated blood samples using the NucleoSpin Blood kit (Macherey-Nagel, Düren, Germany) according to the manufacturer’s instructions.

PCR protocols were designed according to manufacturers’ instructions (Supplementary Table ST8). In general, the total reaction volume was 12.5 µL for all master mixes. Next generation sequencing of PCR amplicons was done on the Illumina MiSeq sequencer using a standard flow cell and v2 500 cycle PE sequencing chemistry. The Illumina Nextera XT kit was used for library preparation following the standard protocol.

### 4.4. Data Analysis and SNP Calling

As described previously [19], the quality of raw sequencing data was checked using FastQC (v0.11.5) [30]. The raw reads were preprocessed using Trimmomatic (v0.36) [31] with the following settings: (a) very low qualities (Phred < 3) were removed from both the 5’ and 3’ ends, (b) low quality bases from the 3’ end were removed using a sliding window approach where we required average the Phred score of four consecutive bases to be at least 5, (c) reads shorter than 35 bp after the preprocessing were discarded, and (d) unpaired reads were discarded as well. The preprocessed reads longer than 150 bp were mapped to a reference sequence using BWA MEM (v0.7.5a) [32]. Alignments were post-processed using SAMtools (v1.4). Alignments were discarded if (a) at least one read from a pair was unmapped, (b) reads were not mapped in a proper pair, (c) secondary alignments were detected, d) non-primary alignments were detected, or e) multi-mapped reads were detected. Additional filtering was performed using NGSUtils/bamutils removeclipping and filter (v0.5.9) [33]. Alignments with more than 5% soft-clipped bases and alignments with more than 10% mismatches (calculated from the mapped read length) were discarded. An additional post-filtering requirement was a minimal read aligned length of 70 bp. Remaining alignments were indel realigned using the GATK (v3.5) [34] and duplicates were removed using Picard tools (v2.1.0) [35]. The average coverage was at least 100x in all the samples in this study. The resulting alignment quality and statistics were checked using the Qualimap (v2.1.3) [36] and custom R (v3.4.1) scripts.

Single nucleotide variants (both SNPs and Indels) were called using the GATK HaplotypeCaller (v3.5) [37], SAMtools (v1.4), and VarScan2 (v2.3.9) [38] according to published best practices recommend by the developers of the tools. GATK (v3.5) base recalibration was applied during the variant calls. Raw variants were hard filtered using the recommended filters for each tool. After the variant calls, an overlap between the tools was created, and high confidence variants were extracted when there was an overlap of at least 2 tools. The number of bases at each variant site was obtained using bam-readcount (v0.8.0) [39], where only alignments with MAPQ at least 10 and Phred base quality of at least 15 were counted. The genotype information was extracted directly from the variant calls from individual tools using VCFtools (v0.1.13) [40]. Merged VCF files (VCFtools) were used for the visual inspection of variants. SNP haplotypes found within the same NGS reads described previously [19] were also analyzed.

### 4.5. Parameters of Population Diversity

For all identified SNPs and/or haplotypes, allele/haplotype frequencies and numbers of reference allele homozygotes/heterozygotes/variant allele homozygotes were calculated for the whole cohort and for every breed studied individually. Chi-square and Fisher’s exact tests were used for assessing differences in allele frequencies between breeds. The level of statistical significance was set at *p* < 0.01. Only SNPs and haplotypes with minor allele frequencies (MAFs) higher than 0.1 were used.

Pairwise FST between each pair of populations and analysis of molecular variance (AMOVA) were estimated using GenAIEx (v6.51) [41]. To infer population structure, Principal coordinate analysis (PCoA-using GenAlEx) and the model-based Bayesian method in STRUCTURE (v2.3.4) software [42] were used. The admixture model with correlated allele frequencies was adopted for this purpose. Each parameter set was analyzed with five replicates for K = 1 to K = 6 and all runs were performed with 10,000 burn-in periods and 50,000 MCMC repeats after burn-in. The optimum K value was assessed based on delta K values from Structure Harvester [43]. Clustering Markov Packager Across K (CLUMPAK) was used for the summation and graphical illustration of the results obtained by STRUCTURE [44].

FST-based comparisons were made for all breed groups, including an overall comparison of all candidate genes analyzed, along with comparisons made for genes associated with fecal shedding at *p*_corrected_ < 0.01, at *p*_uncorrected_ < 0.01 and at *p* > 0.05 in our previous study [19]. For genes associated with FCoV-shedding, differences in allele frequencies and rates of homozygosity between clusters defined by PCoA and Structure were determined using Chi square and Fisher’s exact tests.

## 5. Conclusions

Using a set of candidate markers potentially associated with FCoV infections, interbreed differences in MAFs were observed. However, no clear distinction of individual breeds was identified by PCoA and STRUCTURE analyses. No clustering into groups potentially differing in their resistance/susceptibility to FCoV infection was observed; all markers clustered across breeds.

Analyses of molecular variance revealed that most of the variation observed in the markers studied was due to variation among individuals, while only a minor part could be explained by variation observed among populations.

PCoA and STRUCTURE analyses based on two genes associated with fecal FCoV shedding patterns in our previous study, *NCR1* and *SLX4IP*, identified three clusters differing significantly in frequencies of alleles associated with shedding phenotypes.

## Figures and Tables

**Table 1:** Interbreed comparisons of allele frequencies.

Gene	*CRH*	*CRHR1*	*CRHBP*	*PRF1*	*GZMA*	*GZMB*	*IFNL*	*IFNLR*
Numbers of interbreed differences	11	12	12	11	13	14	9	13
**Gene**	***NCR1***	***NCR2***	***NCR3***	***LAMP5***	***SNX5***	***SLX4IP***	***PAK5***	***PCSK***
Numbers of interbreed differences	14	14	6	8	8	10	7	14

**Table 2:** Pairwise FST values based on: all genes analyzed (left)/genes associated with FCoV shedding at *p*_corrected_ < 0.01 (right).

Domestic Shorthair	Maine Coon	British Shorthair	Bengal	Russian Blue	Norwegian Forest	
0	0.153	0.106	0.142	0.247	0.037	Domestic Shorthair
0.057	0	0.193	0.240	0.215	0.199	Maine Coon
0.090	0.098	0	0.286	0.180	0.039	British Shorthair
0.190	0.236	0.225	0	0.413	0.249	Bengal
0.199	0.230	0.209	0.355	0	0.232	Russian Blue
0.116	0.161	0.101	0.190	0.225	0	Norwegian Forest

**Table 3:** Overall differences in allelic frequencies and homozygosity between the clusters identified by PCoA expressed as numbers of SNPs, for which statistically significant differences were observed.

Allele/Genotype Frequency	All SNPs				Associated SNPs			
Gene	Total	Cluster 1 vs. 2	Cluster 1 vs. 3	Cluster 2 vs. 3	Total	Cluster 1 vs. 2	Cluster 1 vs. 3	Cluster 2 vs. 3
*NCR1* (*p* < 0.01)	38	13	17	11	1	1	1	1
*SLX4IP* (*p* < 0.05)	6	0	0	1	3	0	0	2
Both genes altogether	44	13	17	12	4	1	1	3
**Homozygosity**	**All SNPs**				**Associated SNPs**			
Gene	Total	Cluster 1 vs. 2	Cluster 1 vs. 3	Cluster 2 vs. 3	Total	Cluster 1 vs. 2	Cluster 1 vs. 3	Cluster 2 vs. 3
*NCR1* (*p* < 0.01)	38	6	0	10	1	0	1	1
*SLX4IP* (*p* < 0.05)	6	0	0	1	3	0	0	0
Both genes altogether	44	6	0	11	4	0	1	1

**Table 4:** Haplotypes differing in their frequencies between breeds: *NCR1* haplotypes (left)/*SLX4IP* haplotypes (right).

Breeds	Bengal	British Shorthair	Domestic Shorthair	Maine Coon	Norwegian Forest	Russian Blue
Bengal	--	0	0	1	0	0
British Shorthair	1	--	0	1	0	0
Domestic Shorthair	0	1	--	0	0	0
Maine Coon	0	1	0	--	1	1
Norwegian Forest	1	1	0	1	--	0
Russian Blue	1	1	1	1	1	--

## Data Availability

Data not contained in Supplementary Materials are available in the Mendeley repository at: https://data.mendeley.com/datasets/pk7zrd5266/draft?preview=1 accessed on 3 May 2021.

## Supplementary Materials

The following are available online at https://www.mdpi.com/article/10.3390/pathogens10060778/s1, Supplementary Table ST1: Polymorphisms; Supplementary Table ST2: Inter-breed comparisons, allele frequency; Supplementary Table ST3: Inter-breed comparisons, haplotype frequency; Supplementary Table ST4: List of breeds; Supplementary Table ST5: Candidate genes; Supplementary Table ST6: Designed primers; Supplementary Table ST7: Amplified genes and regions; Supplementary Table ST8: Polymerases used for PCR amplifications; Supplementary Material SM1: All SNPs data, population characteristics; Supplementary Material SM2: PCoA, STRUCTURE, two-gene combinations; Supplementary Material SM3: Cluster analyses (SNPs); Supplementary Material SM4: Haplotypes; Supplementary material SM5: Cluster analyses (haplotypes).
